# Supplementary material for: Reproductive skew affects social information use
Source: R Soc Open Sci. 2019 Jul 3;6(7):182084. doi: 10.1098/rsos.182084 (PMC6689588; doi:10.1098/rsos.182084)
Supplement: ESM [file rsos182084supp1.pdf]

Reproductive skew affects social information use

Supplementary Information

Our supplementary information consist of three parts that provide additional results to the simulation in the main text. These regard: changes in foraging (A), changes in reproduction (B), and changes in learning (C).

A Changes in Foraging

A.1 Increased population density

In the results for figure 2 of the main text, we explain a mechanism that allows social learning to evolve in environments with evenly distributed resources and slow resource turnover. This result only holds where population density is low. Simulations in the main text had $M = 1000$ patches and $N = 1000$ individuals. Given that individuals only forage 80% of the time there are on average always fewer foragers than patches. When we reduce the number of patches and force individuals to share patches (figure S1) we find that the advantage of social learning at the above-mentioned conditions disappears and individual learning dominates at high reproductive skew and evenly distributed resources. This is because here it is less likely for a social learner to enter a patch with only one other individual and successfully displace them, or enter an unoccupied patch.

B Changes in Reproduction

B.1 Limiting case – only evaluating previous round for fitness proxy F

In the main text, we described how the fitness proxy was calculated using payoffs from the previous $\delta = 5$ rounds. To show our qualitative results do not depend on this chosen window size we ran simulations with $\delta = 1$, i.e. where only payoffs from the previous round were evaluated (figure S2). The results are qualitatively similar to those shown in figure 4 of the main text. However, the effect we described for figure 2 that for slow changing environments and evenly distributed resources social learning evolves even at high reproductive skew (lower left in figure S2a,c) is stronger here.

B.2 Reproduction relative to the fitness proxy F

In the main text, we presented results for simulations with the 'minimum income' criterion, i.e. individuals in the top β proportion of the population are equally likely to reproduce. Below are the results for simulations with the 'relative income' criterion, i.e. the probability for an individual i within the top β proportion of the population to reproduce is proportional to its fitness proxy F_i. Because some individuals in the mating pool are more likely to reproduce than others, reproductive skew was greater for the same value of β in the relative income than in the minimum income criterion. This results

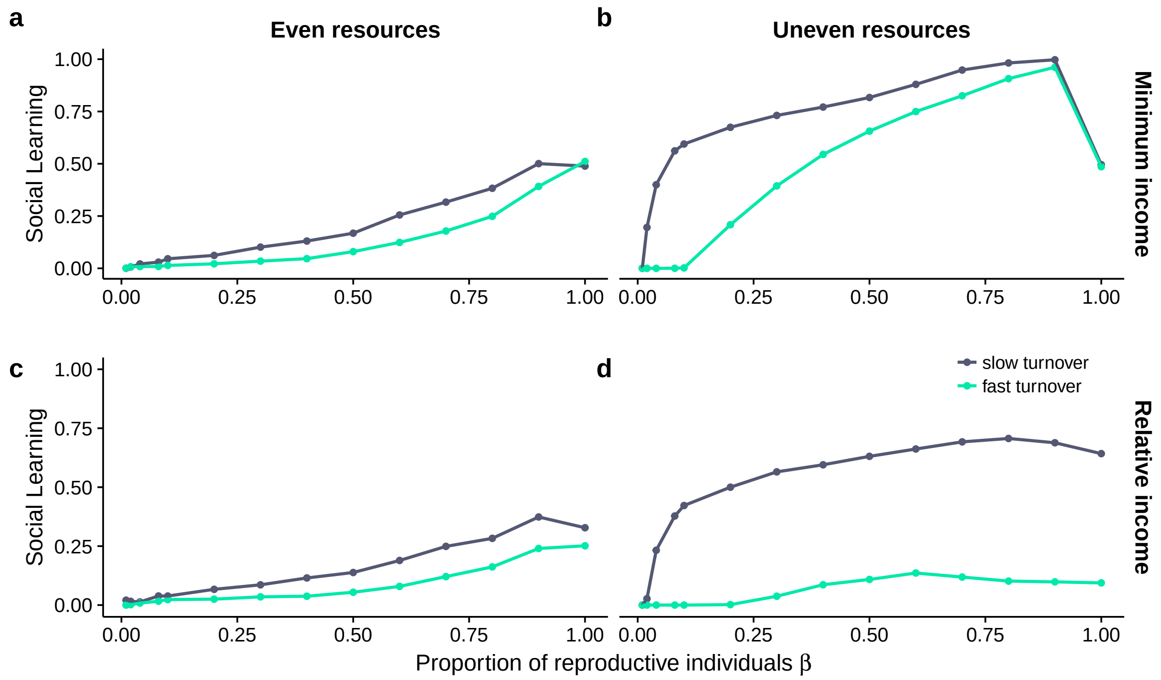

Figure S1: Results for simulations with different environments, income criteria and high population density (1000 individuals, 100 patches). The results for unevenly distributed resources are largely unchanged from those shown in figure 3. Environmental conditions are identical to those in figure 3.

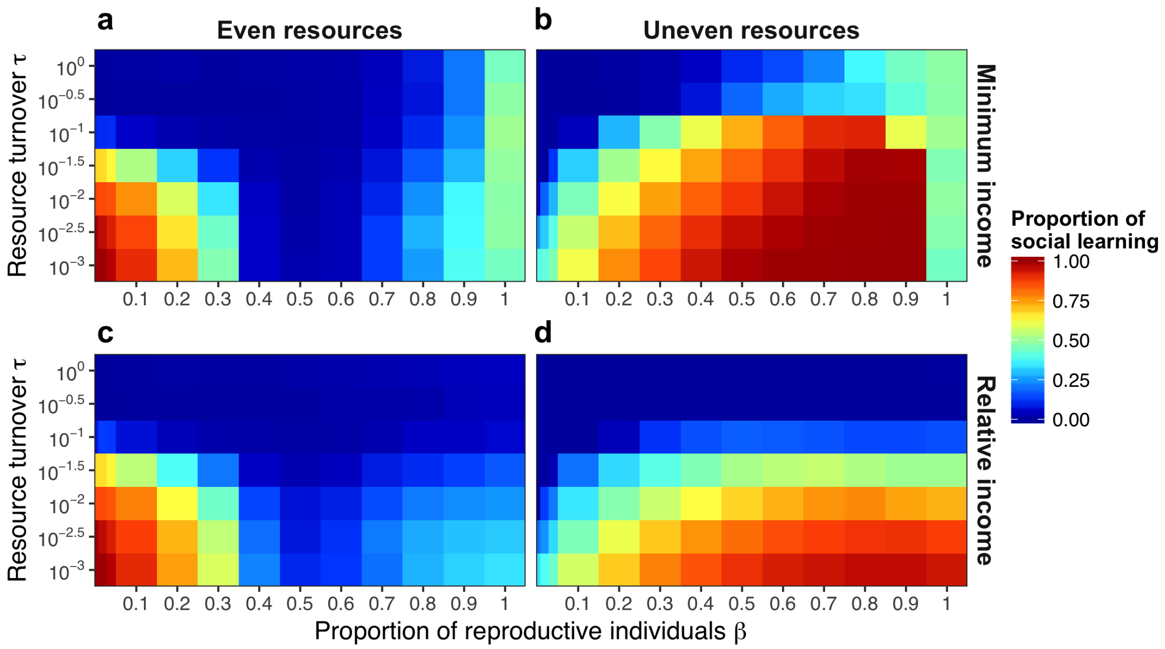

Figure S2: The results for simulations where the fitness proxy is based only on the resources collected in the previous round ($\delta = 1$).

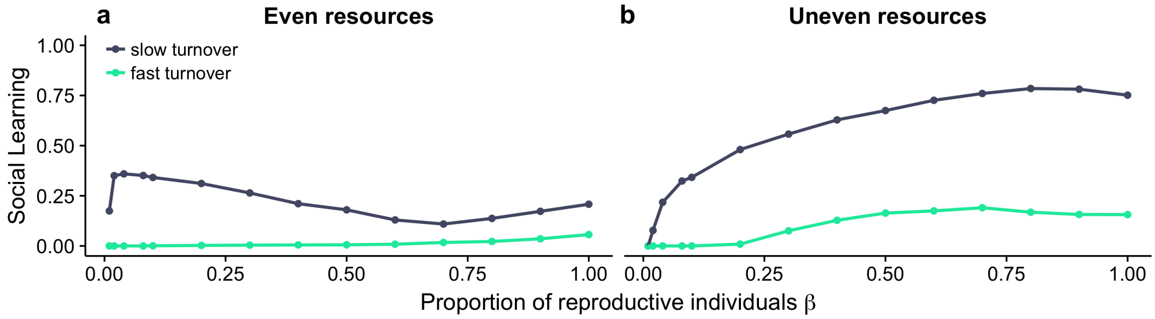

Figure S3: Evolving proportions of social learning for different environments and the relative income scenario. As for the results in figure 3 in the main text, where reproductive skew is highest (small values of β) individuals rely almost exclusively on individual learning, apart from slowly changing, evenly distributed resources. At low reproductive skew (large values of β) there is more social learning. In addition to reproductive skew, the proportion of social learning also depends on the resource turnover and the resource distribution. There is more social learning when resources are unevenly distributed and environmental turnover is slow. Environmental conditions are identical to those in figure 3.

in overall more individual learning especially for environments with unevenly distributed resources (compare figure 4b and S4b).

B.3 Simulations with sexual reproduction and $p_{\mathbf{min}} = 1$

In the main text, we assume that males and females only differ in their reproductive skew, i.e. males experience higher and females lower reproductive skew. Here, we add a further prerequisite for females to be able to reproduce by requiring a minimum of $p_{\text{min}} = 1$ resources collected over the previous two turns. We included this requirement to reflect that female foraging strategies often need to avoid high income variations and thus avoid potentially detrimental energetic shortfall to their offspring.

C Changes in learning

C.1 Mixed learning strategies and asexual reproduction

In the main text, we restrict results to simulations with pure learning, i.e. an individual is either a complete individual ($\alpha = 0$) or a complete social learner ($\alpha = 1$). Here we show results for simulations where social learning propensity can take on any value between 0 and 1 ($\alpha \in [0, 1]$), i.e. an individual with $\alpha = 0.25$ has a 25% chance to learn socially and a 75% chance to learn individually when entering a learning turn.

The simple form of mutation as described in the main text, where $\alpha' = 1 - \alpha$, is here replaced with the following rule. For simulations with asexual reproduction and mixed learning, mutation leads to a new α that is randomly chosen from a normal distribution $N(\alpha, 0.1)$ but bounded on $[0, 1]$. The

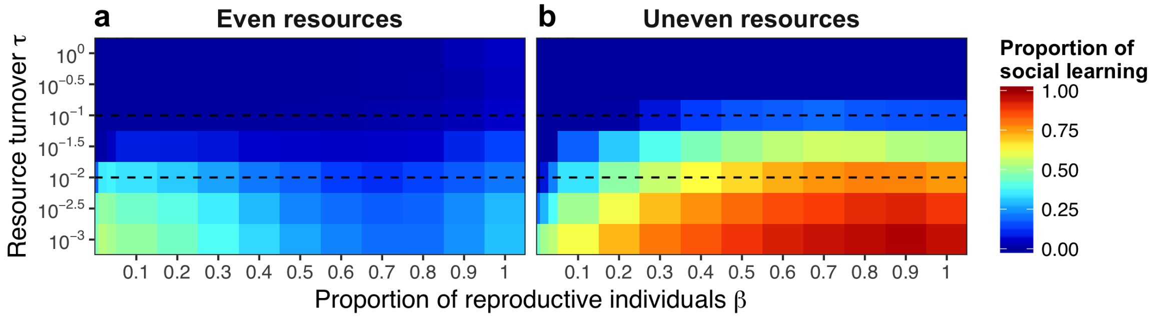

Figure S4: Environmental predictability and reproductive skew affect social learning under different resource distributions. Where resources are unevenly distributed (b) reliance on social learning increases as reproductive skew and environmental turnover decrease. Where resources are evenly distributed and frequently changing (a) individuals almost exclusively rely on individual learning. However, for very slow environmental turnover social learning can evolve even at high reproductive skew. Dashed lines mark high and low resource turnover rates shown in figure S3.

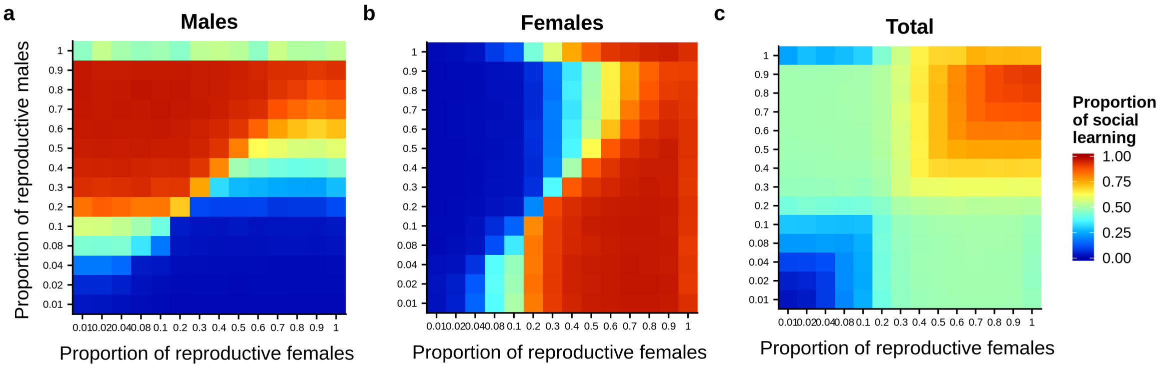

Figure S5: In contrast to the main text, here we show results from simulations where females need to collect at least $p_{\text{min}} = 1$ amount of resources over the previous two rounds, and where β can take any value for both sexes. Below the diagonal females experience weaker reproductive skew than males ($\beta_f > \beta_m$), as analogous to the results reported in the main text. Results above the diagonal represent simulations where females experience stronger reproductive skew than males ($\beta_f < \beta_m$). Because males do not have to collect a minimum of resources to reproduce there is no selection on their learning at $\beta_m = 1$, thus males approach 50% social learning (comparable to the results for females in figure 5B, where $p_{\text{min}} = 0$). However, due to the requirement of $p_{\text{min}} = 1$ females do not approach 50% social learning at $\beta_f = 1$ but instead are relying more on social learning.

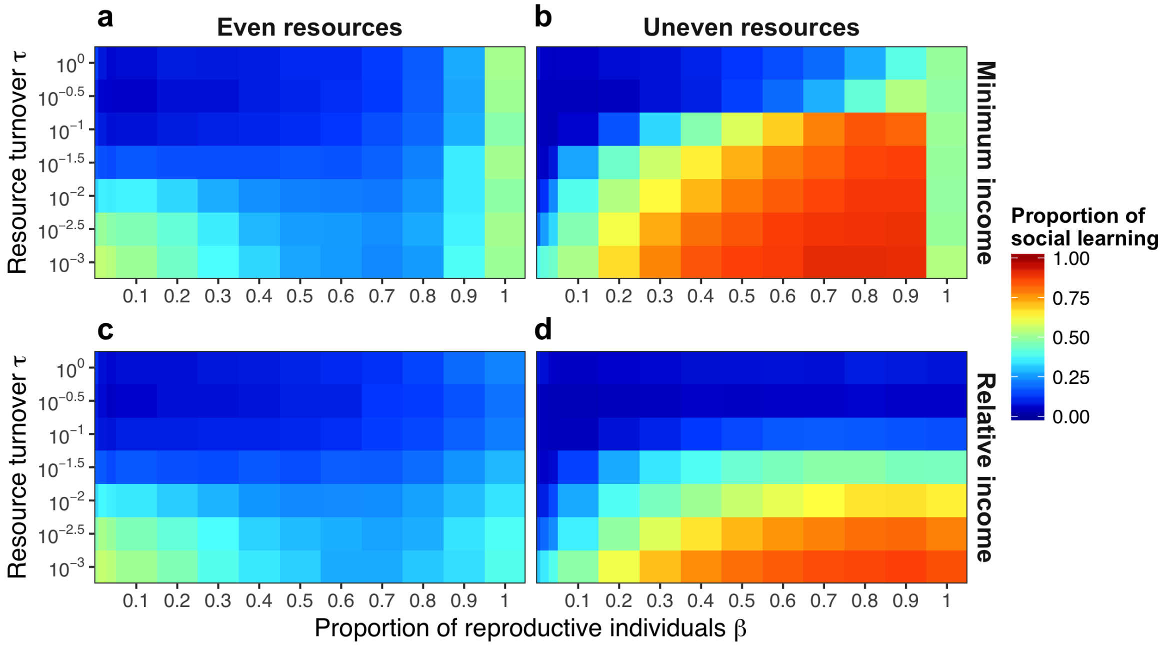

Figure S6: Evolution of social learning relative to reproductive skew β and for different environmental regimes when learning is mixed. Environmental conditions are identical to those in figure 3 from the main text.

results for mixed learning as shown in Fig S2 are qualitatively similar to figure 3 in the main text, but extremes are less pronounced, which has been shown previously in a similar model [35].

C.2 Mixed learning strategies and sexual reproduction

Furthermore, we ran simulations with sexual reproduction and mixed learning (figure S7). In simulations with sexual reproduction an individual's propensity to learn socially corresponds to the social learning propensity inherited from its same-sex parent ($\alpha \in [0, 1]$). For mixed learning α can take any value between 0 and 1. Mutation occurs with probability $\mu = 1/N$, whereby a new value is drawn from a normal distribution centred around the initial value $N(0, 0.01)$ (values are bounded on [0,1]). To initialise simulations with sexual reproduction and mixed learning we randomly assigned values for α drawn from a uniform distribution $U(0, 1)$ to males and females.

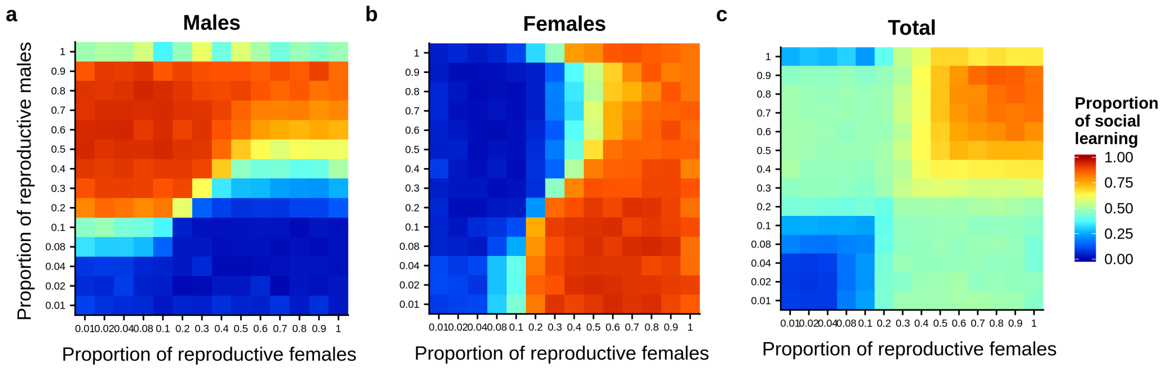

Figure S7: Results for simulations with sexual reproduction, mixed learning, and $p_{\text{min}} = 1$. The results are qualitatively similar to simulations with pure learning (compare figure S5). Environmental parameters are identical to those reported in figure 5.
